# SEOM clinical guideline for the management of cutaneous melanoma (2020)

**DOI:** 10.1007/s12094-020-02539-9

**Published:** 2021-03-02

**Authors:** M. Majem, J. L. Manzano, I. Marquez-Rodas, K. Mujika, E. Muñoz-Couselo, E. Pérez-Ruiz, L. de la Cruz-Merino, E. Espinosa, M. Gonzalez-Cao, A. Berrocal

**Affiliations:** 1 Department of Medical Oncology, Hospital de la Santa Creu i Sant Pau, c/Sant Antoni Maria Claret 167, 08025 Barcelona, Spain; 2 Department of Medical Oncology, H. Germans Trias i Pujol, Catalan Institute of Oncology, ICO-Badalona, Badalona, Spain; 3 Department of Medical Oncology, Instituto de Investigación Sanitaria Gregorio Marañón and CIBERONC, Madrid, Spain; 4 Department of Medical Oncology, UGC de Oncología de Gipuzkoa, OSI Donostialdea-Onkologikoa, Guipúzcoa, Spain; 5 Department of Medical Oncology, Vall d’Hebron Institute of Oncology (VHIO), Hospital Vall d’Hebron Barcelona, Barcelona, Spain; 6 Department of Medical Oncology, Hospital Costa del Sol and UGC Oncol, Instituto de Investigación Biomédica de Málaga (IBIMA), Hospital Universitario Regional Virgen Victoria, Málaga, Spain; 7 Department of Medical Oncology, Hospital Universitario Virgen Macarena, Seville, Spain; 8 Medicine Department, Universidad de Sevilla, Seville, Spain; 9 Department of Medical Oncology, Hospital Universitario La Paz, CIBERONC, Madrid, Spain; 10 Oncology Department (IOR), Hospital Dexeus, Barcelona, Spain; 11 Department of Medical Oncology, Consorcio Hospital General Universitario de Valencia, Valencia, Spain

**Keywords:** Melanoma, Adjuvant treatment metastatic treatment, Staging

## Abstract

Melanoma affects about 6000 patients a year in Spain. A group of medical oncologists from Spanish Society of Medical Oncology (SEOM) and Spanish Multidisciplinary Melanoma Group (GEM) has designed these guidelines to homogenize the management of these patients. The diagnosis must be histological and determination of BRAF status has to be performed in patients with stage ≥ III. Stage I–III resectable melanomas will be treated surgically. In patients with stage III melanoma, adjuvant treatment with immunotherapy or targeted therapy is also recommended. Patients with unresectable or metastatic melanoma will receive treatment with immunotherapy or targeted therapy, the optimal sequence of these treatments remains unclear. Brain metastases require a separate consideration, since, in addition to systemic treatment, they may require local treatment. Patients must be followed up closely to receive or change treatment as soon as their previous clinical condition changes, since multiple therapeutic options are available.

## Methodology

The authors have reviewed the published clinical guidelines, as well as clinical trials from which the aspects referred to in these guidelines can be concluded. Each author has been responsible for reviewing a part of the guideline that has been shared and discussed among all the authors to reach a consensus. Finally, the degrees of evidence and recommendation have been established based on the recommendations for the development of guidelines [1, 2].

## Incidence and epidemiology

The annual incidence of melanoma in Europe is < 10–25/100.000, in the US is 20–30/100.000 and in Australia 50–60/100.000 [3]. It is expected an increase in the next years, and therefore, there is a need to improve prevention and early diagnosis measures [4]. In Spain, an increase in the melanoma mortality rate was observed in the last decades of the twentieth century; however, later, stabilization was observed in women and a decrease in middle age and young men [5, 6]. In 2020, the estimated new cases of melanoma in Spain are 6179 [7].

Ultraviolet radiation is the most important risk factor, especially if intermittent sun exposure, particularly early in life [8]. Individuals with skin types I and II have the highest risk of developing melanoma, as well as individuals with a high numbers of typical nevi, large congenital nevi, and atypical nevi [9, 10]. About 10% of melanomas occur in patients with familial history of melanoma and can present a germline mutation; CDKN2A is the main gene involved [11]. Physical protection is the best prevention of melanoma, and regular use of sunscreen reduces the incidence of cutaneous melanoma (level of evidence 1, grade of recommendation A) [12].

## Diagnosis and pathology

Clinical analysis of suspicious lesions includes three aspects: (1) ABCD rule (Asymmetry, Border irregularities, Color heterogeneity, and Dynamics or evolution in the color, size, or elevation), (2) the ugly duckling sign (the lesion is different from the rest in the same patient), and (3) chronological analysis of changes: assessment of rapid growth in a previous lesion [13]. Dermatoscopy by an experienced physician is recommended for the diagnosis of pigmented lesions (Level of evidence 1b, grade of recommendation A) [14]. Whole-body photography and digital dermatoscopy are useful in people at high risk of melanoma, especially in early diagnosis (Level of evidence 2b, grade of recommendation B) [15]. Reflectance confocal microscopy can be helpful in lesions difficult to diagnose by visual inspection and dermatoscopy (Level of evidence 2b, grade of recommendation B) [16]. All suspicious lesions must be confirmed histologically by excisional biopsy following the eighth edition of the American Joint Committee on Cancer (AJCC 8th edition) [17].

### Molecular testing

Determination of BRAF V600 status is mandatory in patients with resectable or unresectable stage III or IV melanoma (Level of evidence 1, grade of recommendation A). Determination of C-KIT and NRAS status is recommended in BRAF wild-type patients (Level of evidence 2, grade of recommendation C) [18, 19]. A new melanoma subtype with NF1 mutation has recently been defined, but its clinical implications are unknown [20]. Programmed death ligand 1 (PD-L1) expression can be tested in resectable or unresectable stage III and IV, although its determination is not mandatory, since negative cases can respond to anti-PD-1 treatments.

### Staging (Table 1)

TNM staging in melanoma includes physical examination of the entire body. In pT1b–pT4b melanoma, ultrasound (US) for locoregional lymph-node metastasis, and/or computed tomography (CT) or positron emission tomography (PET) scans and brain magnetic resonance imaging (MRI), is recommended for proper tumor assessment (Level of evidence 3, grade of recommendation A) [17].

**Table 1:** Melanoma staging AJCC 8th edition

T category	Thickness	Ulceration status
TX: Primary tumor thickness cannot be assessed (e.g., diagnosis by curettage)	Not applicable	Not applicable
T0: No evidence of primary tumor (e.g., unknown primary or completely regressed melanoma)	Not applicable	Not applicable
Tis (melanoma in situ)	Not applicable	Not applicable
T1	≤ 1.0 mm	Unknown or unspecified
T1a	< 0.8 mm	Without ulceration
T1b	< 0.8 mm	With ulceration
	0.8–1.0 mm	With or without ulceration
T2	> 1.0–2.0 mm	Unknown or unspecified
T2a	> 1.0–2.0 mm	Without ulceration
T2b	> 1.0–2.0 mm	With ulceration
T3	> 2.0–4.0 mm	Unknown or unspecified
T3a	> 2.0–4.0 mm	Without ulceration
T3b	> 2.0–4.0 mm	With ulceration
T4	> 4.0 mm	Unknown or unspecified
T4a	> 4.0 mm	Without ulceration
T4b	> 4.0 mm	With ulceration

N category	No. of tumor-involved regional lymph nodes	Presence of in-transit, satellite, and/or microsatellite metastases
NX	Regional nodes not assessed (e.g., sentinel lymph-node biopsy not performed, regional nodes previously removed for another reason)	No
	Exception: pathological N category is not required for T1 melanomas, use clinical N information	
N0	No regional metastases detected	No
N1	One tumor-involved node or any number of in-transit, satellite, and/or microsatellite metastases with no tumor-involved nodes	
N1a	One clinically occult (i.e., detected by SLN biopsy)	No
N1b	One clinically detected	No
N1c	No regional lymph-node disease	Yes
N2	Two or three tumor-involved nodes or any number of in-transit, satellite, and/or microsatellite metastases with one tumor-involved node	
N2a	Two or three clinically occult (i.e., detected by SLN biopsy)	No
N2b	Two or three, at least one of which was clinically detected	No
N2c	One clinically occult or clinically detected	Yes
N3	Four or more tumor-involved nodes or any number of in-transit, satellite, and/or microsatellite metastases with two or more tumor-involved nodes, or any number of matted nodes without or with in-transit, satellite, and/or microsatellite metastases	
N3a	Four or more clinically occult (i.e., detected by SLN biopsy)	No
N3b	Four or more, at least one of which was clinically detected, or the presence of any number of matted nodes	No
N3c	Two or more clinically occult or clinically detected and/or presence of any number of matted nodes	Yes

M category	Anatomic site	LDH level
M0	No evidence of distant metastasis	Not applicable
M1	Evidence of distant metastasis	See below
M1a	Distant metastasis to skin, soft tissue including muscle, and/or nonregional lymph node	Not recorded or unspecified
M1a(0)		Not elevated
M1a (1)		Elevated
M1b	Distant metastasis to lung with or without M1a sites of disease	Not recorded or unspecified
M1b(0)		Not elevated
M1b (1)		Elevated
M1c	Distant metastasis to non-CNS visceral sites with or without M1a or M1b sites of disease	Not recorded or unspecified
M1c(0)		Not elevated
M1c (1)		Elevated
M1d	Distant metastasis to CNS with or without M1a, M1b, or M1c sites of disease	Not recorded or unspecified
M1d(0)		Not elevated
M1d (1)		Elevated

T	N	M	Stage group
Tis	N0	M0	0
T1a	N0	M0	IA
T1b–T2a	N0	M0	IB
T2b–T3a	N0	M0	IIA
T3b–T4a	N0	M0	IIB
T4b	N0	M0	IIC
Any T, Tis	≥N1	M0	III
Any T	Any N	M1	IV

## Treatment of localized disease

### Treatment of primary tumors

**Fig. 1:**
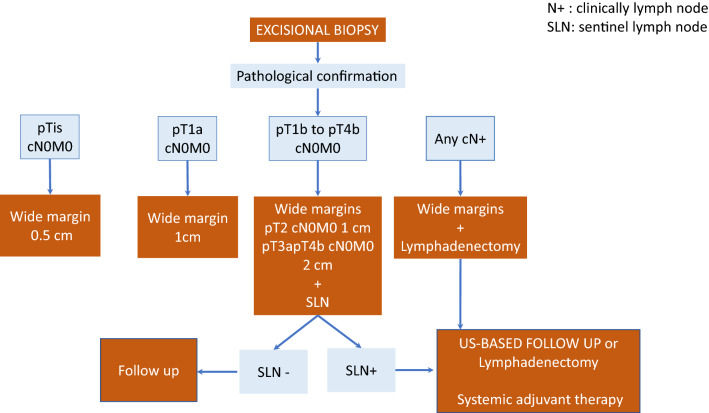
Treatment algorithm for primary tumors and regional lymph notes

Excisional biopsy with a 2 mm lateral margin and deep subcutaneous margin is indicated for any suspicious lesion (Level of evidence 1a, grade of recommendation A). Upon pathological confirmation of the diagnosis, definitive surgery with wide margins is performed. The deep margin should extend to the fascia, whereas lateral margins will depend on Breslow thickness: 0.5 cm for in situ melanomas, 1 cm for tumors with thickness of up to 2 mm, and 2 cm for > 2 mm (Level of evidence 1b, grade of recommendation A) [21]. Figure 1 shows the treatment algorithm for primary tumors.

Sentinel lymph-node biopsy is recommended for melanomas with Breslow > 0.8 mm of thickness or < 0.8 mm with ulceration, i.e., melanomas with stage ≥ IB of the AJCC 8th edition classification (Level of evidence 2a, grade of recommendation A) [22–24].

Complete lymph-node dissection in patients with positive sentinel lymph nodes carries severe morbidity and has shown no impact on survival compared with US-based follow-up (Level of evidence 1b, grade of recommendation A) [25, 26]. For this reason, complete dissection has been abandoned as routine practice. However, the procedure is recommended in the case of clinically detected regional lymph-node metastases (Level of evidence 4, grade of recommendation C) [27].

Resection of satellite or in-transit metastases is associated with a high risk of local and regional progression. With the advent of effective systemic therapies, this strategy could only be considered in highly selected cases (Level of evidence 4, grade of recommendation C).

### Adjuvant therapy

#### Adjuvant radiotherapy

Adjuvant radiotherapy in the local tumor can be considered in cases of inadequate resection margins of lentigo maligna when further resection is not advisable (Level of evidence 3b, grade of recommendation B. The role of radiotherapy for in-transit metastasis has not been established (Level of evidence 5, grade of recommendation D). Nodal adjuvant radiotherapy reduces the risk of regional recurrence after resection of palpable regional lymph nodes or extracapsular extension [28]. However, it increases the risk of regional toxicity—particularly lymphedema—with no impact on overall survival, so it is not longer routinely recommended (Level of evidence 2b, grade of recommendation B) [29]. Radiotherapy may be discussed in resected head and neck melanoma with palpable lymph nodes, where local control is critical and has a lower risk of lymphedema.

#### Adjuvant systemic therapy

For patients with complete resection of a cutaneous melanoma, to recommend adjuvant systemic therapy depends upon the risk of disease recurrence, based on the stage at diagnosis, along with a consideration of patient age, comorbidity, and personal preferences. For patients with stage III melanoma, adjuvant immunotherapy with nivolumab or pembrolizumab is indicated; and dabrafenib and trametinib are an alternative for patients with *BRAF* V600 mutation. In patients with completely resected stage IV, adjuvant nivolumab is also indicated.

Nivolumab for 1 year prolonged RFS at 4-year follow-up compared with ipilimumab (52% vs. 41%, HR: 0.71, *p* < 0.0001, respectively) in patients with completely resected stage IIIB, IIIC, or IV. Efficacy was observed both in BRAF mutant and wild-type patients. In the first report of overall survival, with fewer events than expected, OS rates were similar in both treatment groups (78% Nivolumab and 77% ipilimumab), taking into account that more patients treated with ipilimumab received subsequent therapy, including immunotherapy (57% with ipilimumab and 49% with nivolumab) [30, 31].

Pembrolizumab for 1 year has also shown prolonged RFS compared with placebo in patients with completely resected stage III melanoma at 3.5-year follow-up (59′8% vs. 41′4%, HR 0,59, *p* < 0.0001, respectively). Efficacy was observed both in BRAF mutant and wild-type patients. Pembrolizumab also decreased the 3.5-year incidence of distant metastases as first recurrence and locoregional recurrence only. The S1404 phase III trial compares pembrolizumab with high-dose interferon or high-dose ipilimumab in patients with completely resected high-risk stage III–IVA disease; accrual is complete, and results are pending [32, 33].

Dabrafenib plus trametinib for 1 year showed a longer RFS compared with placebo in completely resected stage III *BRAF* V600 mutant melanoma at 5 year follow-up (52% vs. 36%, HR 0.51, 95% CI 0.42–0.61), irrespective of baseline factors. Overall survival, at a median follow-up of 2.8 years, was prolonged with the targeted therapy, but remains immature [34–37].

**Fig. 2:**
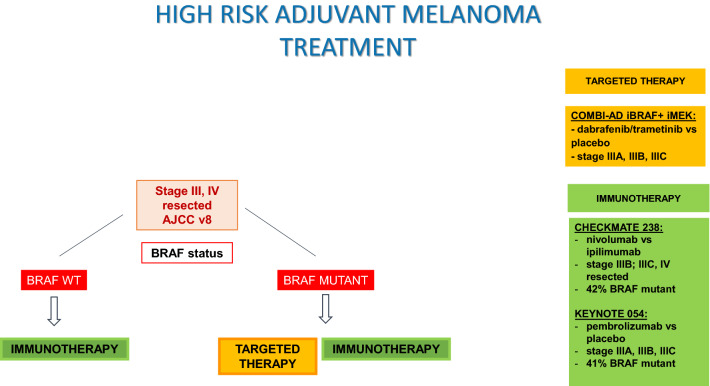
Adjuvant treatment algorithm in high-risk melanoma

In summary, in patients with completely resected stage III or IV melanoma, adjuvant immunotherapy and targeted therapies have shown to improve RFS (Level of evidence 1, grade of recommendation A). Although in all these pivotal clinical trials, lymphadenectomy was required as an inclusion criteria, the impact of avoiding a lymphadenectomy on the results of adjuvant treatment is unknown, leaving the decision whether or not performing it before in the context of a discussion with the patient (Level of evidence 3, grade of recommendation C). Finally, stage IIIA AJCC v7 patients were included in COMBI-AD and Keynote 054 trials with the inclusion criteria of sentinel lymph nodes > 1 mm; regulatory approvals of FDA, EMA, and AEMPS admit to extend the use of adjuvant systemic treatment to with anti-PD-1 antibodies all stage III, including stage IIIA, but there is no clear evidence of its use in IIIA with < 1 mm (Level of evidence 3, grade of recommendation B–C). Selection of a specific agent depends largely on *BRAF* mutation status and toxicity profiles (Level of evidence 3–4, grade of recommendation B). Figure 2 shows adjuvant treatment algorithm in high-risk melanoma.

For patients who relapse with metastatic disease after initial adjuvant therapy, options include treatment with an alternative active systemic therapy or inclusion in a clinical trial (Level of evidence 3, grade of recommendation C) [38].

## Treatment of advanced disease

### Oligometastatic disease

Some stage IV patients present with a resectable, oligometastatic disease and surgical excision or stereotactic radiosurgery (SRS) of solitary metastases should be considered whenever feasible. One-third of patients with resected metastasis may become long-term survivors (Level of evidence 2b, grade of recommendation B) [39]. Surgery may be the preferred option for selected patients if feasible, preferentially combined with adjuvant systemic therapies as previously commented.

### Systemic treatment of unresectable advanced/m1 disease

#### Treatment options in first line

##### Immunotherapy

Immunotherapy based on immune checkpoint inhibitors has demonstrated its superiority over chemotherapy in terms of response, PFS and OS in first-line treatment (Level of evidence 1, grade of recommendation A). This option, unlike that occurs with targeted therapy against BRAF, is not based on any biomarker such as the expression of the programmed death-ligand 1 (PD-L1) (Level of evidence I, grade of recommendation B) or tumor mutational burden (TMB) (Level of evidence IV, grade of recommendation C). Ipilimumab, an anti-CTLA-4 (cytotoxic T-lymphocyte-associated protein 4) antibody, was the first treatment to show an improvement in overall survival in patients with metastatic melanoma [40, 41]. This option has been exchanged in favor to PD-1 inhibitors such as nivolumab [42, 43], pembrolizumab [44, 45], or the combination of ipilimumab plus nivolumab [46, 47]. Table 2 summarizes the main characteristics of the pivotal trials of immunotherapy in advanced melanoma.

**Table 2:** Main characteristics of the pivotal trials of immunotherapy in advanced melanoma

Treatment	Considerations	PFS (median months 95% IC)	OS (median months, 95% IC)	Update overall survival (%)
Nivolumab [3, 4] CheckMate 066	BRAF WT patients Control arm: DTIC Protocol amendment: cross over nivolumab	5.1 (3.5–10.8)	NR (survival rate at 1 year 72.9% (65.5–78.9)	51.2% at 3 years
Pembrolizumab [5, 6] KEYNOTE-006	Control arm: ipilimumab 34% second-line treatment 36% BRAF mutant	4.1 (2.9 a 6.9)*	NR (survival rate at 1 year 68.4%)	32.7 (24.5–41.6) at 5 years**
Ipilimumab + nivolumab [7, 8] CheckMate 067	Control arm: ipilimumab 31.5% BRAF mutant	11.5 (8.9–16.7)	64% survival rate at 2 years	58% at 3 years; 52% at 5 years

*NR* not reached, *NC* unknown

*At dose 10 mg/kg every 3 weeks

**Combined pembrolizumab groups

The clinical criteria that can help us to choose the best first-line treatment option with immune checkpoint inhibitors (monotherapy or combination) or the duration of treatment are not yet established. Different studies have proposed several clinical markers such as LDH (lactate dehydrogenase), lymphocytes, leukocytes, or eosinophils count that could predict the response to ICI, but data are inconclusive (Level of evidence IV, grade of recommendation C) [48].

On the other hand, there is other type of immunotherapy such as Talimogene Laherparepvec, an intralesional virotherapy that has shown an improvement in durable response rate, overall survival, and loco-regional control respect to GM-CSF in patients with injectable lesions and unresectable stage IIIB–C or IV melanoma, specially when used as first-line therapy (Level of evidence 1, grade of recommendation B) [49].

##### Targeted therapy

Currently, there are three different combinations of BRAF and MEK inhibitors based in four randomized phase III clinical trials that improve both progression-free survival and overall survival in comparison with BRAF inhibitors alone (Table 3) [50, 51]. The combination of BRAF and MEK inhibitors should be the therapy of choice when targeted therapy is considered, unless absolute contraindication for MEK inhibitors is present (Level of evidence 1, grade of recommendation A).

**Table 3:** Randomized trials with of BRAF and MEK inhibitors in advanced BRAF mutant melanoma

Combination	Progression-free survival (median months, 95% IC)	Overall survival (median months, 95% IC)	Patients alive at 3, 4, and 5 years (%)	References
Dabrafenib + trametinib	11.1 (9.5–12.8)	25.9 (22.6–31.5)	44,37,34	[1]
Vemurafenib + cobimetinib	12.6 (9.5–14.7)	22.5 (20.3–28.8)	38.5, 34.7, NA	[2]
Encorafenib + binimetinib	14.9 (11.0–20.2)	33.6 (24.4–39.2)	47, 39, NA	[3]

There are certain subgroups that of poor prognosis, being the most important factors an elevated LDH, ECOG > 1 and high tumor burden (expressed in number of organs involved) [50, 52]. However, a combination of BRAF and MEK inhibitor would be the first option over monotherapy (Level of evidence 2, grade of recommendation B).

Direct comparison among the three different combos does not exist, and similar efficacy with small differences in the toxicity profile has been described. When selecting the combination, patient preferences, drug availability, and efficiency criteria should be taken into account (Level of evidence 4, grade of recommendation C).

##### First-line selection in BRAFm

The best treatment sequence for BRAF mutant patients is unknown, since no direct comparison exists. First-line decision between targeted therapies or immunotherapy is currently being studied in prospective trials (SECOMBIT, NCT02631447) to define the best sequencing combination treatment in terms of OS, the primary efficacy variable. Meanwhile, the decision should be based on patient’s profile (comorbidities, ECOG, symptoms, and life expectancy) and on melanoma characteristics (tumor burden, site of metastasis, and level of LDH) [52, 53] (Level of evidence 2, grade of recommendation B).

Most studies have demonstrated a higher number of events during the first 12 months with immunotherapy than with targeted therapy and, by contrast, patients with immunotherapy have better survival beyond the first year [50, 54]. With all the aforementioned, it can be advised that in those patients where the first months of treatment can be administered safely (for example, those who will not progress quickly, or where immediate response is not required due to involvement of an organ or its function) would be good candidates for immunotherapy, reserving targeted therapy for later lines (Level of evidence 2, grade of recommendation B).

#### Treatment options in second line

Treatment options in second line depend on the treatment administered in the first line, as well as, on the mutational status. For the BRAF wild-type patients, options for a second-line treatment are limited, and inclusion in clinical trials is a priority. When the first line was an anti-PD-1 antibody, ipilimumab (Level of evidence 2, grade of recommendation B) and the combination of nivolumab and ipilimumab (Level of evidence 4, grade of recommendation B) are two valid options [55]. Finally, chemotherapy (Dacarbazine and Temozolomide) can be considered for patients who exhausted other options (Level of evidence 2, grade of recommendation D).

In the BRAF mutant population treated with an anti-PD-1 antibody in first line, the combination of BRAF and MEK inhibitors is the preferred option. Although their activity has not been prospectively studied after progression to anti-PD-1, it seems to be similar to the first line of treatment in terms of response (Level of evidence 2, grade of recommendation A) [56]. Data from the Columbus Study, where BRAF mutant patients may have previously received immunotherapy, showed that these patients also benefited from the combination (Level of evidence 2, grade of recommendation A) [57].

In the BRAF mutant population treated with the combination of BRAF and MEK inhibitors in first line, anti-PD-1 antibody and the combination of nivolumab and ipilimumab are valid options.

#### Treatment beyond progression

Treatment beyond progression might be an option in selected patients both on targeted as well as on immunotherapy based on retrospective data [58]. No randomized data are available at this time. Treatment beyond progression is generally not recommended unless there is a suspicion of pseudo-progression, bearing in mind that the analysis of these data is subject to many biases related to patient status.

#### Management of brain metastasis

It is estimated that up to 50–60% of metastatic melanoma patients will develop brain metastasis. First, a stepwise and multidisciplinary approach is highly recommended to design an individualized plan for every patient (Level of evidence 4, grade of recommendation A) [59]. In an effort to classify patients into similar prognostic groups, some key clinical factors such as Karnofsky Performance Score (KPS), number of brain metastases, extracranial metastases, age, and BRAF status constitute the basis of the specific graded prognostic assessment (GPA) index for melanoma (Melanoma-mol GPA) [60] and could be extremely helpful to guide clinical decision-making (Level of evidence 3, grade of recommendation B).

Among locoregional strategies, surgery must be considered to treat symptomatic and large brain metastasis, especially in the case of solitary brain metastasis and when a pathological and/or molecular diagnosis is needed (Level of evidence 4, grade of recommendation B) [61]. SRS is generally recommended in patients with 1–4 brain metastases with less than 3–4 cm (Level of evidence 3, grade of recommendation A), although the role of SRS has been tested for up to 15 brain metastasis [61]. Adjuvant SRS yields the same overall survival but with fewer declines in cognitive function, so SRS on the surgical cavity and not WBRT is recommended after excision of brain metastases (Level of evidence 1b, grade of recommendation A) [62]. Whole brain radiation therapy (WBRT) is discouraged except in the palliative setting when other options are not feasible (Level of evidence 4, grade of recommendation C) [59].

When locoregional strategies are discarded, systemic therapies must be considered. At this point, two main clinical situations emerge. In asymptomatic, previously untreated brain metastasis patients, combination immunotherapy with nivolumab and ipilimumab has demonstrated outstanding results with intracranial response rates ranging from 46 to 57% and median PFS and DOR not reached with a median follow-up of 20.6 months [63]. Pembrolizumab, nivolumab, and ipilimumab as single therapies also show some degree of activity (ORR around 20%) [64]. In BRAF mutant patients, BRAF and MEK inhibitors obtain high intracranial responses, which rises up to 58% with the combination of dabrafenib and trametinib [65]. However, these responses seem to be shorter than those obtained in extracranial sites. Aforementioned results make reasonable advising nivolumab plus ipilimumab as the preferred first-line treatment for patients with asymptomatic brain metastasis not amenable to surgery or SRS, irrespective of BRAF status, whenever possible (Level of evidence 3, grade of recommendation A). In BRAF-mutated patients, combination of BRAF and MEK inhibitors remains a good alternative option in first and second lines (Level of evidence 3, grade of recommendation B).

Unfortunately, symptomatic and/or previously treated patients obtain poorer results, especially with immunotherapy, and new approaches are eagerly needed in this setting [59].

### Follow-up

On average, 20–30% of early stage melanoma patients will develop a recurrence within 5 years [66]. For stage I–II melanomas, recurrences will be in 50% of cases at regional lymph nodes, 30% as distant metastasis and 20% will be local relapses or in-transit metastasis. For stage III melanoma, up to 95% occur during the first 3 years of follow-up [66], and up to 50% will be distant recurrences, 25% regional and 25% local relapses. About 2–10% will have a second primary melanoma, most of them during the first year after initial diagnosis [67].

Self-examination is an essential component of the follow-up and can lead to early recognition of recurrences and new melanomas [68]. Patients should receive instructions on self-examination (Level of evidence 3, grade of recommendation B). Over the last few years, several skin cancer detection applications for smartphones have been developed through analysis of artificial intelligence algorithms. Further studies are needed before implementing these techniques in the general population (Level of evidence 4, grade of recommendation C).

Physical examination has proven to be an effective procedure for early recurrence detection [68] and should be performed in all melanoma patients during follow-up (Level of evidence 2, grade of recommendation A). Physical exam must include skin and nodes exam. Total body photography, sequential digital dermatoscopy imaging, and reflectance confocal microscopy, must be helpful in patients with a high number of moles or presence of clinical atypical nevi (Level of evidence 3, grade of recommendation A). For stage I–IIA melanomas, the frequency of physical exams should be, at least, annually, life-long, but it depends on the risk factors of each patient (Level of evidence 3, grade of recommendation A). For patients with stage IIB–IV melanomas, physical examinations should be performed every 3–6 months during the first 2 years, and then every 3–6 months for 3 years, and after 5 years, they could be done annually (Level of evidence 3, grade of recommendation A).

Routine blood testing is optional, as few recurrences are detected by increased LDH and S-100 levels [68] (Level of evidence 4, grade of recommendation C). Liquid biopsy for melanoma screening using the determination of BRAF mutation in cfDNA from blood or other body fluids is a promising technique, but it is not routinely indicated yet (Level of evidence 4, grade of recommendation C).

Lymph-node sonography has proven to be the most sensitive and most specific procedure for the detection of locoregional lymph-node metastases [69]. In patients with stage IIC–III melanomas, lymph-node sonography of regional areas must be performed regularly, and it should be performed every 4 months during the first 2 years, and every 6 months during the next 3 years, especially in patients with positive sentinel lymph nodes without lymph-node dissection (Grade of recommendation A; level of evidence 1).

General recommendation about imaging procedures is not possible, since no prospective studies have assessed if early recurrence detection impacts in the overall survival. Some studies in IIC–III melanoma patients have demonstrated that an extensive follow-up including CT body scan and brain MRI detects almost 50%, and 8% of recurrences [70, 71], so imaging follow up every 3 months is recommended (Grade of recommendation B; level of evidence 3). Routine follow-up with PET–CT is not recommended, although some studies have demonstrated a higher sensitivity for detecting distant metastases in the extremities (Grade of recommendation C; level of evidence 4) [72]. For earlier stages I–IIA where the risk of relapse is lower, radiological follow-up with body CT scan and brain MRI is optional (Grade of recommendation C; level of evidence 4).

## Summary of recommendations and evidence

**Table:** 

	Level of evidence	Grade of recommendation
Physical protection and regular use of sunscreen reduce the incidence of cutaneous melanoma	1	A
*Diagnosis and pathology*	1b	A
Dermatoscopy by an experienced physician is recommended for the diagnosis of pigmented lesions		
Whole-body photography and digital dermatoscopy are useful in people at high risk of melanoma, especially in early diagnosis	2b	B
Determination of BRAF V600 status is mandatory in patients with resectable or unresectable stage III or IV melanoma	1	A
*Staging*		
In pT1b–pT4b melanoma, US for locoregional LN metastasis, CT or PET-TC and brain MRI, is recommended	3	A
*Treatment of primary tumor*		
Excisional biopsy is recommended on all suspicious lesions	1a	A
Safety surgical margins should be Breslow-adapted	1b	A
SLN is indicated if Breslow > 0.8 mm or < 0.8 mm with ulceration	2a	A
Routine CLND is not recommended for SLN + patients	1b	A
CLND is recommended for clinically detected regional lymph nodes	4	C
*Adjuvant therapy*		
Adjuvant radiotherapy is not recommended, but it may be considered for selected cases	2b	B
Adjuvant anti-PD-1 treatment with nivolumab and pembrolizumab or targeted therapies with dabrafenib and trametinib are recommended in resected stage III–IV melanoma	1	A
*Oligometastatic disease*		
Surgical excision or SRS of solitary metastases should be considered	2b	B
*Management of advanced disease*		
First line advanced/M1 disease		
Anti-PD-1 treatment (nivolumab or pembrolizumab) or ipilimumab and nivolumab are a standard of care for all patients with advanced melanoma	1	A
Intralesional T-VEC is also an option for unresectable stage IIIB–C or IV	1	C
In BRAF V600 melanoma, BRAF and MEK inhibitors (dabrafenib–trametinib, vemurafenib–cobimetinib, encorafenib–binimetinib) are additional first-line options	1	A
Second line and beyond advanced/M1 disease		
In BRAF wild-type melanoma, ipilimumab and the combination of nivolumab and ipilimumab are two valid options after an anti-PD-1 treatment	2, 4	B
In BRAFV600 melanoma, BRAF and MEK inhibitors are the preferred option after an anti-PD-1 treatment	2	A
In BRAFV600 melanoma treated with BRAF and MEK inhibitors in first line, anti-PD-1 treatment (nivolumab or pembrolizumab) or ipilimumab and nivolumab are valid options	2	A
Chemotherapy can be considered for patients who exhausted other options	2	D
*Brain metastases*		
Multidisciplinary approach is highly recommended	4	A
Melanoma-mol GPA index could help to guide clinical decision	3	B
Surgery must be considered in symptomatic, large BM specially in solitary BM	4	B
SRS is recommended in 1–4 BM metastases with less than 3–4 cm	3	A
*Follow-up*		
Self-examination is recommended in all patients	3	B
Physical examinations is recommended in all patients	2	A
Routine blood testing is optional	4	C
Follow-up with CT body scan and brain MRI in stages IIB–IV is recommended	3	B
Follow-up with CT body scan and brain MRI in stages I–IIA is optional	4	C
Lymph-node US is recommended in SLN-positive patients	1	A

*SLN* sentinel lymph node, *CLND* complete lymph-node resection, *SRS* stereotactic radiosurgery, *BM* brain metastasis
